# Supplementary material for: Deactivation of ligand-receptor interactions enhancing lymphocyte infiltration drives melanoma resistance to Immune Checkpoint Blockade
Source: bioRxiv. 2023 Sep 22:2023.09.20.558683. Preprint. [Version 1] doi: 10.1101/2023.09.20.558683 (PMC10602042; doi:10.1101/2023.09.20.558683)
Supplement: 1 [file NIHPP2023.09.20.558683V1-supplement-1.pdf]

bioRxiv preprint doi: https://doi.org/10.1101/2023.09.20.558683; this version posted September 22, 2023. The copyright holder for this preprint (which was not certified by peer review) is the author/funder. This article is a US Government work. It is not subject to copyright under 17 USC 105 and is also made available for use under a CC0 license.

64.

-

Supplementary figures

bioRxiv preprint doi: https://doi.org/10.1101/2023.09.20.558683; this version posted September 22, 2023. The copyright holder for this preprint (which was not certified by peer review) is the author/funder. This article is a US Government work. It is not subject to copyright under 17 USC 105 and is also made available for use under a CC0 license.

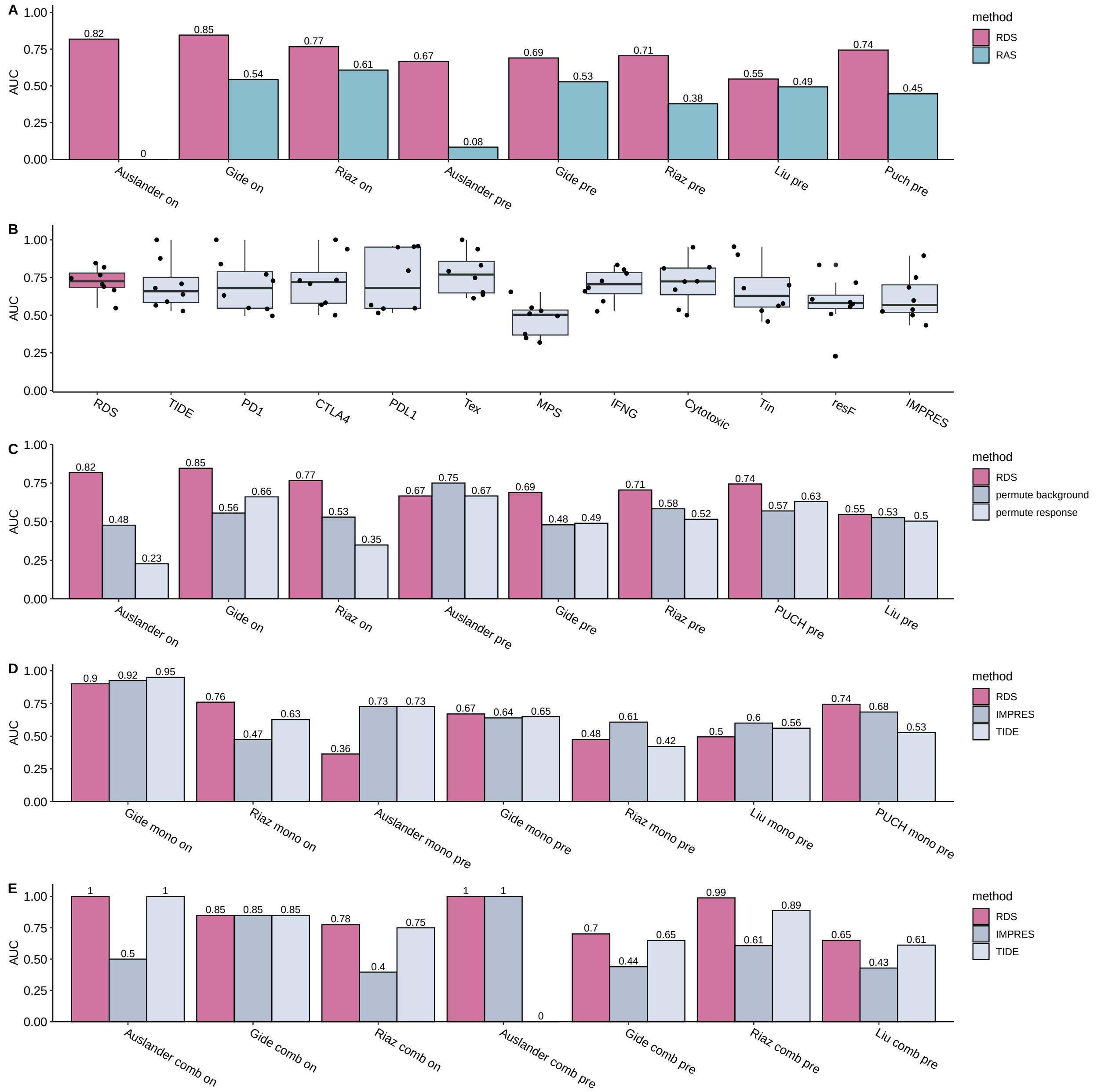

Supplementary Figure 1: (A) Bar plot depicting AUC in classifying responder vs. non-responder melanoma samples between resistance deactivated (RDS) and resistance activated (RAS) interactions. (B) Boxplot depicting the distribution of AUC in classifying responder vs. non-responder samples in all melanoma ICB cohorts between RDS and 10 relevant transcriptomic signatures of ICB and immune response: TIDE (7), *PD1*, *CTLA4*, *PDL1*, T-cell exhaustion (Tex), Melanocytic plasticity score (MPS) (9), IFNG signature (47), Cytotoxic signatures (10), T-cell inflamed GEP (Tin) (48), functional ICB resistance (resF) (11), IMPRES (8). (C) Bar plot depicting AUC in classifying responder vs. non-responder melanoma samples between RDS, RDS when permuting cell-type-specific ligand-receptor interaction profile, and RDS when permuting patient response. (D) Bar plot depicting AUC in classifying responder vs. non-responder melanoma ICB monotherapy (anti-PD1 or anti-CTLA4) samples between RDS, TIDE

bioRxiv preprint doi: https://doi.org/10.1101/2023.09.20.558683; this version posted September 22, 2023. The copyright holder for this preprint (which was not certified by peer review) is the author/funder. This article is a US Government work. It is not subject to copyright under 17 USC 105 and is also made available for use under a CC0 license.

(7), and IMPRES (8). (E) Bar plot depicting AUC in classifying responder vs. non-responder melanoma ICB combination therapy (anti-PD1 with anti-CTLA4) samples between RDS, TIDE (7), and IMPRES (8).

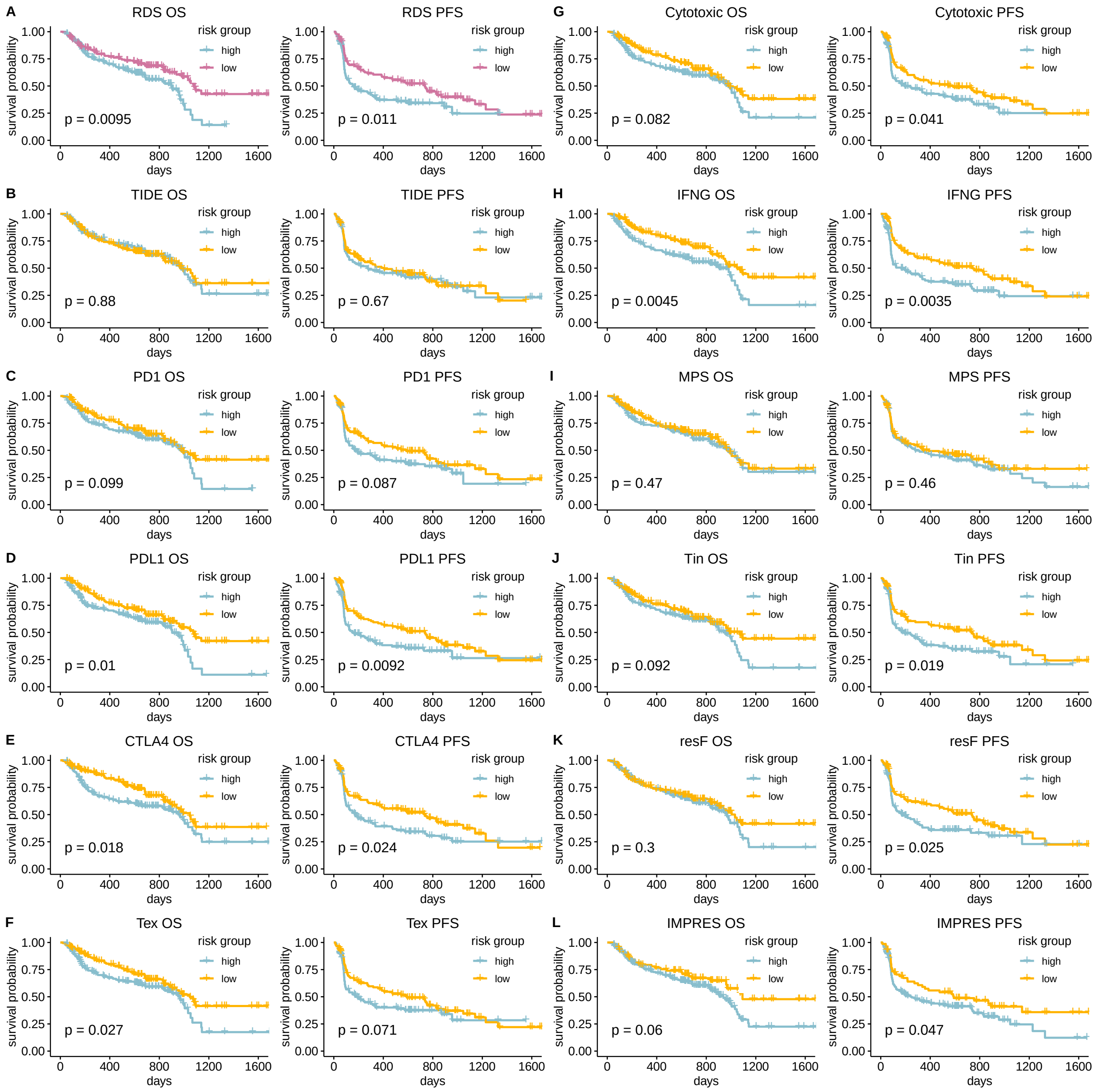

Supplementary Figure 2: Survival stratification performance of RDS score vs. other relevant bulk-transcriptomics signatures of the combined set of pre-treatment melanoma samples receiving immune checkpoint blockade. (A) Kaplan-Meier plots depicting progression free (PFS) and overall survival (OS) differences between the low and high-risk groups defined by the median value of RDS. The significance of survival differences was estimated using the log-rank test. Time on the X-axis is measured in days. (B-L) Kaplan-Meir plots showing PFS and OS differences between low and high-risk groups defined by the median value of relevant transcriptomic signatures. The significance of survival differences was estimated using the log-rank test. Time on the X-axis is measured in days. The signatures

bioRxiv preprint doi: https://doi.org/10.1101/2023.09.20.558683; this version posted September 22, 2023. The copyright holder for this preprint (which was not certified by peer review) is the author/funder. This article is a US Government work. It is not subject to copyright under 17 USC 105 and is also made available for use under a CC0 license.

evaluated in each panel are: (B) TIDE (7), (C) *PD1*, (D) *CTLA4*, (E) *PDL1*, (F) T-cell exhaustion (Tex), (G) Melanocytic plasticity score (MPS) (9), (H) IFNG signature (47), (I) Cytotoxic signatures (10), (J) T-cell inflamed GEP (Tin) (48), (K) functional ICB resistance (resF) (11), (L) IMPRES (8).

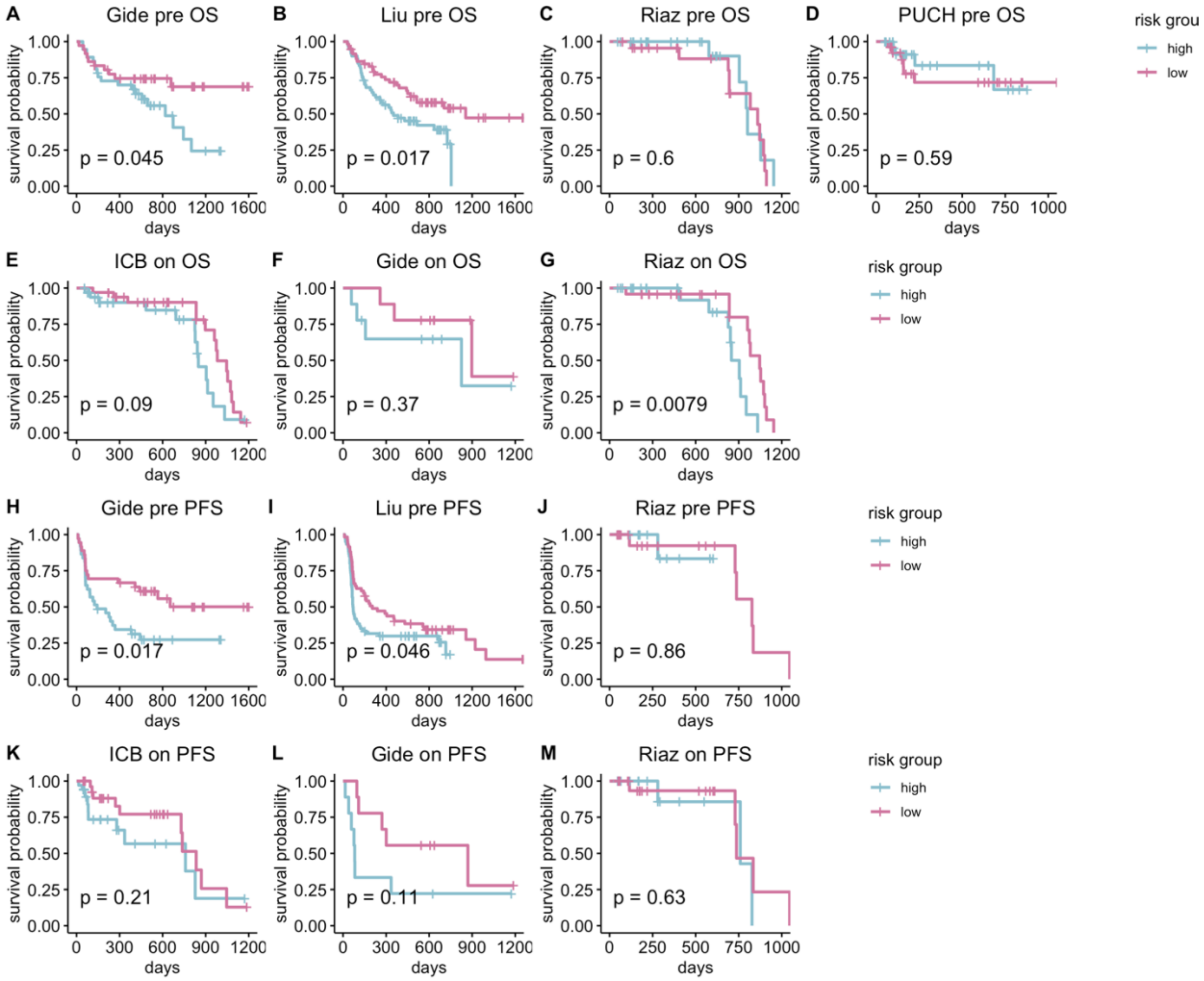

Supplementary Figure 3: Survival stratification performance of RDS for melanoma patients receiving checkpoint therapy. Kaplan-Meir plots showing progression free survival (PFS) and overall survival (OS) differences between low and high-risk groups defined by the median value of RDS. The significance of survival differences was estimated using the log-rank test. Time on the X-axis is measured in days. (A-D) Kaplan-Meir plots depicting OS of individual checkpoint cohorts' pre-treatment patients. (E) Kaplan-Meir plots depicting OS of the combined set on-treatment checkpoint treated patients. (F-G) Kaplan-Meir plots depicting OS of individual checkpoint cohorts' on-treatment patients. (H-J) Kaplan-Meir plots depicting PFS of individual checkpoint cohorts' pre-treatment patients. (K) Kaplan-Meir plots depicting PFS of the combined set on-treatment checkpoint treated patients. (L-M) Kaplan-Meir plots depicting PFS of individual checkpoint cohorts' on-treatment patients.

bioRxiv preprint doi: https://doi.org/10.1101/2023.09.20.558683; this version posted September 22, 2023. The copyright holder for this preprint (which was not certified by peer review) is the author/funder. This article is a US Government work. It is not subject to copyright under 17 USC 105 and is also made available for use under a CC0 license.

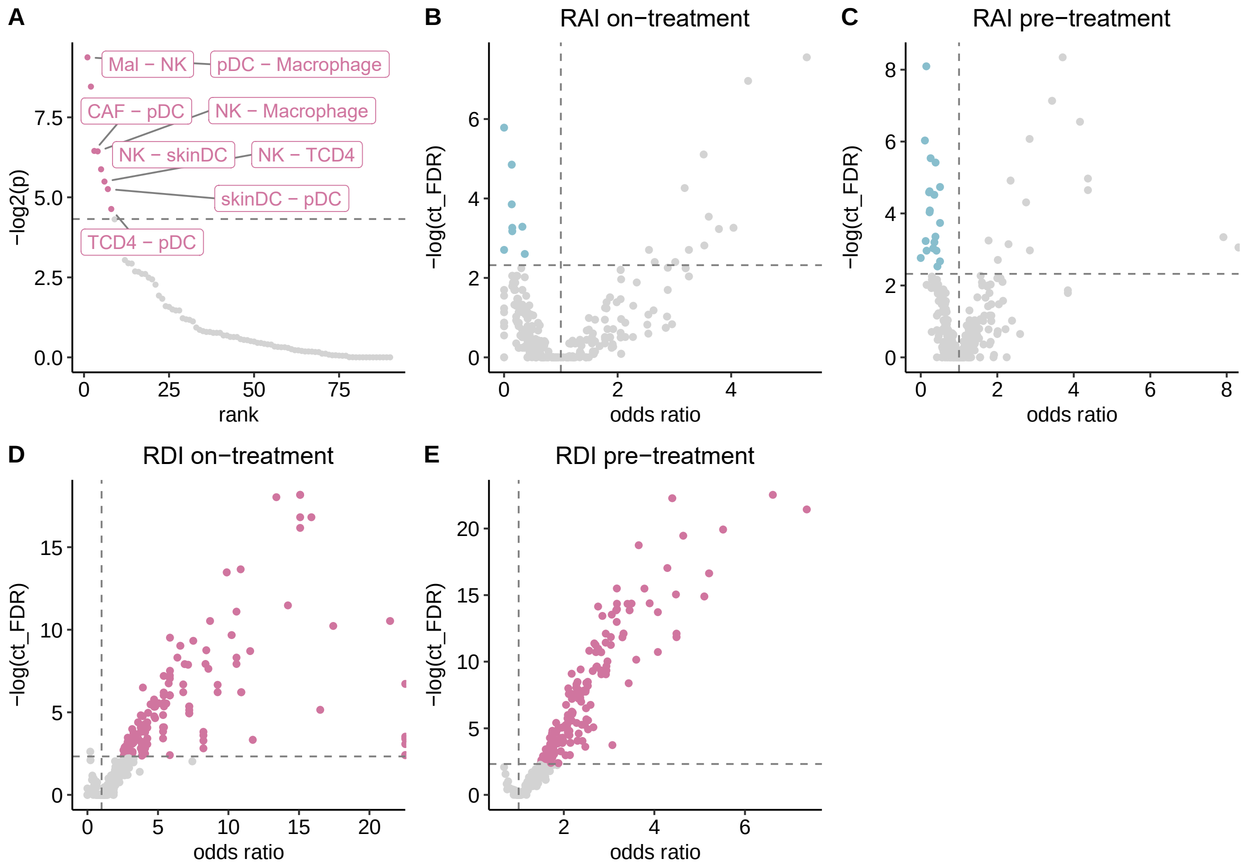

Supplementary Figure 4: (A) Enrichment analysis depicting top ranked ligand cell-receptor cell pairs enriched within the RDI network. Background were all LIRICS' tumor-immune interactions inferred (N=3776). The Y-axis indicates the p-value from the cell pair enrichment analysis. The X-axis indicates the rank in ascending order starting from the most significantly enriched cell-pair. Cell pairs with p-value < 0.05 (dotted line) were considered significantly enriched and are highlighted in magenta. (B) Enrichment analysis depicting individual active RAIs enrichment in responder compared to non-responder patients in the combined set of on-treatment samples receiving immune checkpoint blockade. The X-axis indicates the odds-ratio of either being enriched in the responder (> 1) compared to non-responder (< 1) samples. The Y-axis indicates the significance (FDR) of individual interactions enrichment in on-treatment responder samples (Fisher's one-sided test). Interactions with an odds-ratio < 1 and FDR < 0.2 per cell type pair are considered significantly activated in non-responder on-treatment samples and are highlighted in blue. (C) Enrichment analysis depicting individual active RAIs enrichment in responder compared to non-responder patients in the combined set of pre-treatment samples receiving immune checkpoint blockade. The X-axis indicates the odds-ratio of either being enriched in the responder (> 1) compared to non-responder (< 1) samples. The Y-axis indicates the significance (FDR) of individual interactions enrichment in pre-treatment responder samples (Fisher's one-sided test). Interactions with an odds-ratio < 1 and FDR < 0.2 per cell type pair are considered significantly activated in non-responder pre-treatment samples and are highlighted in blue. (D) Enrichment analysis depicting individual active RDIs enrichment in responder compared to non-responder patients in the combined set of on-treatment samples receiving immune checkpoint blockade. The X-axis indicates the odds-ratio of either being enriched in the responder (> 1)

bioRxiv preprint doi: https://doi.org/10.1101/2023.09.20.558683; this version posted September 22, 2023. The copyright holder for this preprint (which was not certified by peer review) is the author/funder. This article is a US Government work. It is not subject to copyright under 17 USC 105 and is also made available for use under a CC0 license.

compared to non-responder (< 1) samples. The Y-axis indicates the significance (FDR) of individual interactions enrichment in on-treatment responder samples (Fisher's one-sided test). Interactions with an odds-ratio > 1 and FDR < 0.2 per cell type pair are considered significantly activated in responder on-treatment samples and are highlighted in magenta. (E) Enrichment analysis depicting individual active RDIs enrichment in responder compared to non-responder patients in the combined set of pre-treatment samples receiving immune checkpoint blockade. The X-axis indicates the odds-ratio of either being enriched in the responder (> 1) compared to non-responder (< 1) samples. The Y-axis indicates the significance (FDR) of individual interactions enrichment in pre-treatment responder samples (Fisher's one-sided test). Interactions with an odds-ratio > 1 and FDR < 0.2 per cell type pair are considered significantly activated in responder pre-treatment samples and are highlighted in magenta.

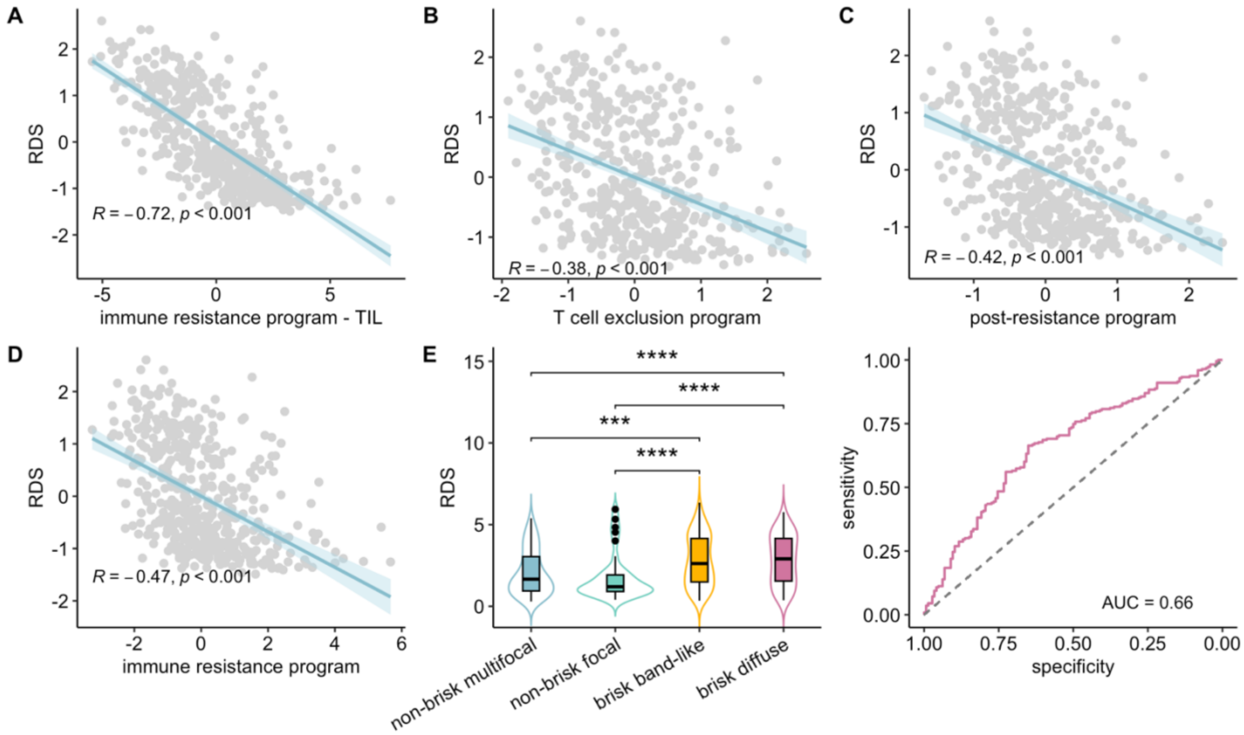

Supplementary Figure 5: (A) Scatter plot depicting the correlation the between RDS and transcriptomic signatures of immune resistance in TCGA-SKCM. The immune resistance program includes the combined set of genes inferred for both the T-cell exclusion and post-resistance programs. The X-axis indicates the immune resistance program adjusted for TIL. The pearson correlation coefficient is 0.72 (p-value < 0.001). (B) Scatter plot depicting the correlation the between RDS and transcriptomic signatures of T-cell exclusion program in TCGA-SKCM. The pearson correlation coefficient is -0.38 (p-value < 0.001). (C) Scatter plot depicting the correlation the between RDS and transcriptomic signatures of post-resistance program in TCGA-SKCM. The pearson correlation coefficient is -0.42 (p-value < 0.001). (D) Scatter plot depicting the correlation the between RDS and transcriptomic signatures of immune resistance program in TCGA-SKCM. The immune resistance program includes the combined set of genes inferred for

bioRxiv preprint doi: https://doi.org/10.1101/2023.09.20.558683; this version posted September 22, 2023. The copyright holder for this preprint (which was not certified by peer review) is the author/funder. This article is a US Government work. It is not subject to copyright under 17 USC 105 and is also made available for use under a CC0 license.

both the T-cell exclusion and post-resistance programs. The pearson correlation coefficient is -0.47 (p-value < 0.001). (E) Boxplot depicting distribution of RDS between non-brisk and brisk subtypes in TCGA-SKCM. (F) ROC curve depicting classifying hot vs. cold tumor niches in TCGA-SKCM using RDS.
